# Suppressors of Cytokine Signaling and Hepatocellular Carcinoma

**DOI:** 10.3390/cancers14102549

**Published:** 2022-05-22

**Authors:** Ryota Masuzaki, Tatsuo Kanda, Reina Sasaki, Naoki Matsumoto, Kazushige Nirei, Masahiro Ogawa, Seth J. Karp, Mitsuhiko Moriyama, Hirofumi Kogure

**Affiliations:** 1 Division of Gastroenterology and Hepatology, Department of Medicine, Nihon University School of Medicine, Itabashi, Tokyo 173-8610, Japan; kanda.tatsuo@nihon-u.ac.jp (T.K.); sasaki.reina@nihon-u.ac.jp (R.S.); matsumoto.naoki@nihon-u.ac.jp (N.M.); nirei.kazushige@nihon-u.ac.jp (K.N.); ogawa.masahiro@nihon-u.ac.jp (M.O.); moriyama.mitsuhiko@nihon-u.ac.jp (M.M.); kogure.hirofumi@nihon-u.ac.jp (H.K.); 2 Department of Surgery, Vanderbilt University Medical Center, Nashville, TN 37232, USA; seth.karp@vumc.org

**Keywords:** hepatocellular carcinoma, suppressor of cytokine signaling, liver regeneration

## Abstract

**Simple Summary:**

Hepatocellular carcinoma (HCC) is a common malignancy worldwide. The HCC generally develops in the liver of patients already suffering from chronic liver disease. There have been significant advances in both the curative and palliative treatment of HCC. Although liver resection is a curative treatment for HCC, its indication is often limited due to an impaired liver function reservoir. There is still a need to understand how to control liver regeneration after resection and find better cancer immunotherapy and anticancer drugs for advanced HCC. Suppressors of cytokine signaling (SOCS) negatively regulate cytokine signaling related to cell proliferation, differentiation, and immune response; therefore, SOCS are thought to play an important role in HCC development and liver regeneration.

**Abstract:**

Cytokines are secreted soluble glycoproteins that regulate cellular growth, proliferation, and differentiation. Suppressors of cytokine signaling (SOCS) proteins negatively regulate cytokine signaling and form a classical negative feedback loop in the signaling pathways. There are eight members of the SOCS family. The SOCS proteins are all comprised of a loosely conserved N-terminal domain, a central Src homology 2 (SH2) domain, and a highly conserved SOCS box at the C-terminus. The role of SOCS proteins has been implicated in the regulation of cytokines and growth factors in liver diseases. The SOCS1 and SOCS3 proteins are involved in immune response and inhibit protective interferon signaling in viral hepatitis. A decreased expression of SOCS3 is associated with advanced stage and poor prognosis of patients with hepatocellular carcinoma (HCC). DNA methylations of SOCS1 and SOCS3 are found in HCC. Precise regulation of liver regeneration is influenced by stimulatory and inhibitory factors after partial hepatectomy (PH), in particular, SOCS2 and SOCS3 are induced at an early time point after PH. Evidence supporting the important role of SOCS signaling during liver regeneration also supports a role of SOCS signaling in HCC. Immuno-oncology drugs are now the first-line therapy for advanced HCC. The SOCS can be potential targets for HCC in terms of cell proliferation, cell differentiation, and immune response. In this literature review, we summarize recent findings of the SOCS family proteins related to HCC and liver diseases.

## 1. Introduction

Hepatocellular carcinoma (HCC) is a common malignancy worldwide, responsible for 5% of all newly diagnosed cancers [1]. Primary liver cancer was ranked sixth for cancer incidence and third for deaths in 2020. Due to the advanced nature at presentation, most cases are incurable and 810,000–830,000 people die every year in the world due to liver cancer [1,2]. Liver cancer was the leading cause of cancer mortality in Mongolia, Thailand, Cambodia, Egypt, Guatemala, and an additional 18 countries, only among men, in 2020 [1]. Despite this grim prognosis, there have been significant advances in both the curative and palliative treatment of HCC, including liver resection, local ablation therapy, radiotherapy, chemotherapy, and liver transplantation [3,4,5,6]. 

One of the major hurdles to performing liver resection for HCC is the need to resect large portions of the liver, leaving insufficient residual tissue to maintain homeostasis. This leads to liver insufficiency and death. A liver transplant is an excellent option for HCC, with an estimated cure rate of 75.8% [7], but this option is limited by low organ supply.

A promising approach for increasing the number of patients that could tolerate resection would be to develop techniques or technologies to increase liver mass either pre- or post-operative, and therefore permit resections that were previously impossible. Makuuchi et al. first reported portal vein embolization of the diseased side of a liver to be resected to induce hypertrophy of the non-diseased remnant liver [8]. In other words, if liver volume could be increased, surgeons could resect more liver, and curative resection would be more widely indicated and achieved. 

To address this important clinical problem, we have focused on the suppressors of the cytokine signaling (SOCS) family because SOCS gene expression levels have been shown to increase early after liver resection in experiments on mice [9,10]. Cytokines regulate major cellular growth and differentiation, including embryonic development, wound healing, immunity, and hematopoiesis. 

**Figure 1:**
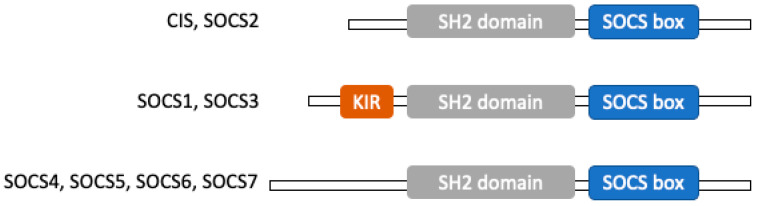
The structure of the SOCS protein. All SOCS family members contain an Src homology 2 (SH2) domain and a segment called the SOCS box located near the C terminal. The SOCS1 and SOCS3 proteins have a unique 12-residue N-terminal kinase inhibitory region (KIR) domain. The KIR domain plays an important role in the interaction with Janus kinase (JAK)/signal transducers and activators of transcription (STAT) pathway. Abbreviation: CIS, cytokine-inducible Src homology 2 domain-containing protein; SOCS, suppressor of cytokine signaling; SH2, Src homology 2; KIR, kinase inhibitory region.

The SOCS family is a group of intracellular proteins related to cytokine downstream signaling, which generally block Janus kinase (JAK)/signal transducers and activators of transcription (STAT) pathway [11,12,13]. To date, eight members of the SOCS family are known. Yoshimura et al. first identified a novel early gene induced in response to several cytokines in 1995 and described it as cytokine-inducible Src homology 2 (SH2) domain-containing protein (CIS) [14]. Next, SOCS1 was reported by three groups in 1997 as a novel JAK regulatory protein [12,15,16]. Proteins SOCS2, SOCS3, SOCS4, SOCS5, SOCS6, and SOCS7 were found in searches of DNA and protein databases. The SOCS family members all contain an Src homology 2 (SH2) domain and a segment called the SOCS box located near the C terminal [17]. Both SOCS2 and CIS show 38% amino-acid sequence similarity, and SOCS1 and SOCS3 have 25% amino-acid sequence similarity [12]. The SOCS1 and SOCS3 have a kinase inhibitory region (KIR) domain [18]. The structure of SOCS protein is shown in Figure 1. 

**Figure 2:**
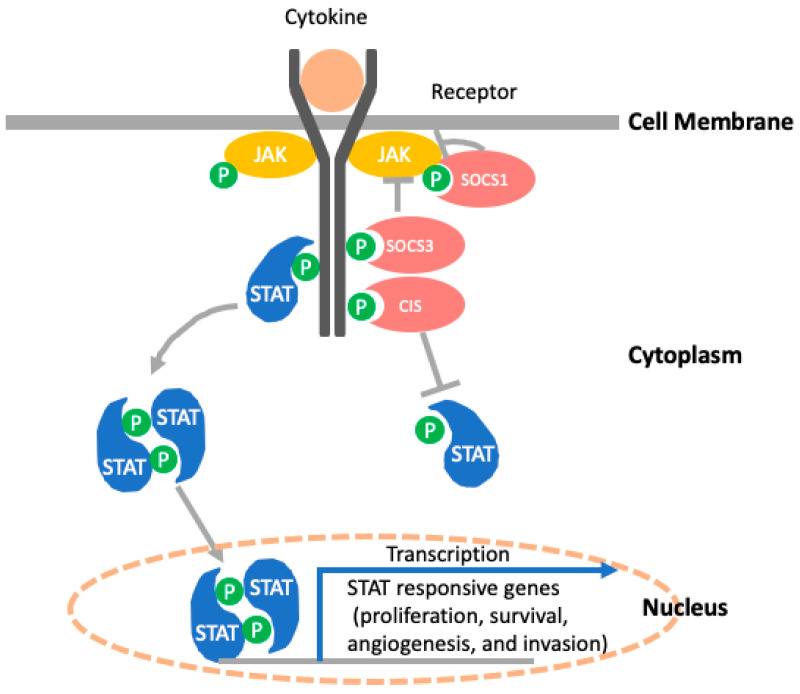
The representative mechanism of suppressor of cytokine signaling (SOCS) proteins. Cytokine binds to specific cytokine receptors and causes receptor dimerization or oligomerization and recruits Janus kinase (JAK)s. Activated JAKs phosphorylate the receptor cytoplasmic domain and assemble signal transducers and activators of transcription (STAT) dimers. The SOCS proteins inhibit cytokine signaling by binding to SOCS or phosphorylated JAKs. Cytokine-inducible Src homology 2 domain-containing protein (CIS) inhibits the recruitment of STAT. Abbreviation: JAK, Janus kinase; SOCS, suppressor of cytokine signaling; STAT, signal transducers and activators of transcription; CIS, cytokine-inducible Src homology 2 domain-containing protein.

The SOCS expression can be induced by cytokine binding to a cognate receptor. The binding results in activation of the JAK/STAT pathway and induces *SOCS* gene transcription [19]. The SOCS family proteins have three mechanisms to inhibit cytokine signaling. The representative mechanism of SOCS is shown in Figure 2. They act as a pseudo-substrate or compete with JAK or STATs for binding sites of activated cytokine receptors. The SOCS box interacts with the adaptor proteins elongin B/C, RING domain-containing protein (RBX2), and the scaffold protein Cullin 5 to recruit E2 ubiquitin-transferase and facilitates the ubiquitination and subsequent proteasomal degradation [20,21,22]. The *SOCS1* mRNA is regulated by *microRNA-155* (*miR-155*) at the post-transcriptional level [23], whereas post-translational regulation of SOCS1 includes phosphorylation by Pim serine/threonine kinases [24,25]. Epigenetic inactivation of SOCS1 is known to be caused by Cp-G island hypermethylation in many types of cancers [26,27,28,29,30].

Hepatitis C virus (HCV) and hepatitis B virus (HBV) infections are well-recognized risk factors for HCC. Nonalcoholic steatohepatitis and other metabolic diseases have been recently identified as risk factor for HCC [4,5,6,31]. The SOCS family proteins are associated with insulin signaling and growth hormone (GH) signaling, which are associated with metabolic syndromes [32]. Several studies have revealed a relationship between the SOCS family proteins and cancer development and prognosis [33]. The SOCS family of proteins are potential key molecules for controlling liver regeneration after liver resection, and moreover, they can be a treatment target for HCC. 

In this literature review, we summarize the relationships between each of the specific SOCS proteins and liver cancer, disease progression, and regeneration. Each SOCS protein and the liver-related signaling pathways are summarized in Table 1.

## 2. SOCS1 and Liver Diseases

The HCC disease usually develops in the liver of patients already suffering from chronic hepatitis with persistent inflammation caused by viruses, alcohol, and/or obesity [3]. The repeating cycle of hepatocyte injury and regeneration results in the accumulation of genetic and epigenetic alterations leading to the activation of oncogenic signaling pathways (such as catenin beta-1 [CTNNB1], nuclear factor-erythroid 2-related factor 2 [NFE2L2], and telomerase reverse transcriptase [TERT]) and inactivation of tumor suppressor pathways (such as TP53, PTEN, SOCS1, and SOCS3) [42,43,44,45].

The SOCS1 protein, the dominant member of the family, functions as a negative regulator in insulin signaling and in the immune response [15,46]. SOCS1-deficient mice are born healthy but die within three weeks after birth with fulminant hepatitis, growth retardation, and thymic atrophy [47]. The role of SOCS1 in liver regeneration was studied using *Socs1*^−/−^*Ifng*^−/−^ mice and the SOCS1-deficient mice displayed significantly faster gain in liver mass as compared with *Ifng*^−/−^ and wild type mice after partial hepatectomy (PH) [34]. Despite the accelerated rate of proliferation, the final restored liver masses of SOCS1-deficient mice were not increased in the study. This indicates liver mass restoration is maintained by SOCS1 and by the other factors. Similar findings were also recognized in our SOCS2 study [9]. 

The SOCS1 protein is often repressed in HCC and the incidence of aberrant methylation in the CpG island of SOCS1 has been reported to be 65% in 26 human primary HCC tumor samples [26]. Moreover, SOCS1 seems to be silenced by methylation and cannot block JAK activation. Okochi et al. reported that 30 of 50 (60%) HCCs had aberrant methylation and that HCC developed in cirrhosis had a significant relationship with SOCS1 methylation [48]. Yoshida et al. investigated the methylation status in the CpG island of the SOCS1 gene in 209 samples of DNA obtained from needle liver biopsy and found that the frequency of methylation correlated with the severity of liver fibrosis [49]. Ko et al. reported SOCS1 gene methylation was more prevalent in HCV-related HCC than HBV-related HCC (84% vs. 55%) [50]. 

MicroRNA has been shown to play important roles in SOCS1 function. The *miR-155* is encoded by a non-coding gene named as *MIR155HG* (formerly known as *BIC)* and is highly inducible in macrophages in response to toll-like receptor (TLR) ligands [51]. The TLR plays a critical role in innate immune responses against microbial pathogens [52]. Bala et al. found increased *miR-155* levels and decreased expression of SOCS1 in Kupffer cells of alcohol-fed mice [53]. Wahid et al. reported that the *SOCS1* gene expression level was positively correlated with liver function tests in chronic hepatitis C patients [54].

Numerous studies have investigated the relationship between interferon (IFN) and viral hepatitis [55,56,57,58]. There are type I, II, and III IFNs. Type I IFNs are a large subgroup of IFNs that helps regulate the activity of the immune system [59]. The IFN-alpha and -beta signaling pathways have been widely studied in HBV and HCV and the drugs have also been used for therapy of patients with chronic hepatitis C and B. Type II IFN is interferon-gamma and binds to different receptors [60]. Type III IFN consists of four IFN lambda, known as IFN-lambda1 (IL-29), IFN-lambda2 (IL-28A), IFN-lambda3 (IL-28B), and IFN-lambda4. IL-28B SNP is significantly associated with a sustained virologic response (SVR) to IFN/ribavirin combination therapy against HCV [61]. The SOCS proteins are thought to inhibit these interferon signaling pathways [62,63]. Song et al. reported that SOCS1 and SOCS3, but not SOCS2, inhibited IFN-alpha- and -gamma-induced antiviral activity [64]. Both SOCS1 and SOCS3 inhibit IFN-alpha-induced expression of antiviral protein oligoadenylate synthetase (2′, 5′-OAS) and myxovirus resistance A (MxA) [35]. Direct acting antivirals (DAAs) are now recognized as the standard of care for chronic hepatitis C patients [65]. Naz et al. compared the expressions of SOCS1 and SOCS3 in DAA- and IFN-treated patients and found SOCS1 and SOCS3 levels of DAA-treated patients were close to healthy patients as compared with IFN-treated patients [66].

## 3. SOCS2 and Liver Diseases

**Figure 3:**
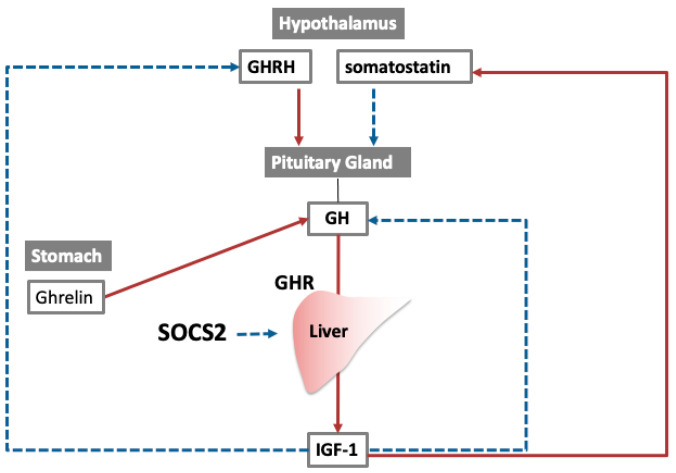
Feedback loop in SOCS2 signaling. Growth hormone (GH) secretion by pituitary gland is regulated by two hormones released from hypothalamus. Insulin-like growth factor (IGF)-1 is secreted from the liver under the influence of GH, and IGF-1 also regulates GH by negative feedback loop. Suppressor of cytokine signaling (SOCS)2 inhibits the IGF-1 secretion by inhibiting Janus kinase (JAK)/signal transducer and activator of transcription (STAT) signaling. Red arrows indicate activation, blue dotted arrows indicate inhibition. Abbreviations: GH, growth hormone; GHR, growth hormone receptor; GHRH, growth hormone releasing hormone; SOCS, suppressor of cytokine signaling; IGF, insulin like growth factor.

The SOCS2 protein acts as a negative regulator in GH signaling through JAK2-STAT5 pathway, and itsdeficiency leads to gigantism [67]. We previously reported the role of SOCS2 in liver regeneration using SOCS2-deficient mice [9]. The *Socs2* mRNA increased 6 h after PH and returned to baseline by 24 h. Loss of SOCS2 led to a significant increase in hepatocyte proliferation at an early time point after PH, but later resulted in a significant decrease in the liver-to-body weight ratio in 7 days. These findings indicate that SOCS2 preserves liver function by limiting the rate of proliferation at an early time point, preventing GH signals via ubiquitination. At later time points, SOCS enhances hepatocyte proliferation by GH release from the pituitary gland. Growth hormone signaling is controlled by other proteins other than SOCS2, such as ghrelin, growth hormone releasing hormone, and somatostatin. (Figure 3). 

Cui et al. found that SOCS2 expression was reduced in HCC tissues as compared with control tissues and the decreased expression was associated with the presence of intrahepatic metastasis and with histologically poorer differentiation [68]. Ren et al. identified SOCS2 as a functional target of miR-196a and miR-196b, and miR-196a and miR-196b expressions were enhanced in HCC tissue and cells [69]. Li et al. developed a prognostic signature based on the Cancer Genome Atlas Project (TCGA) and found that SOCS2, reticulon 3 (RTN3) and beta-ureidopropionase (UPB1) expression levels were independent predictors for the prognosis of HCC [70]. A low level of SOCS2 with a high level of RTN3 had a worse survival outcome as compared with other combinations. They also evaluated the protein level in HCC tissue and found the expression of SOCS2 was decreased in HCC. 

Zadjali et al. investigated the role of SOCS2 in hepatic steatosis using high-fat diet (HFD)-mice [71]. The HFD-fed *Socs2−*/*−* mice exhibited less extensive steatosis and enhanced expression of inflammatory cytokines in the liver. The HFD-fed *Socs2−*/*−* mice also had severe insulin resistance as compared with the wild type mice. On the other hand, HFD-fed SOCS1-deficient mice displayed hepatic steatosis with increased expression of lipogenic genes and had hyperglycemia with insulin resistance [72]. The HFD-fed liver-specific SOCS3-deficient mice also had increased liver fat and insurance resistance [73]. There seem to be distinct differences in the regulation of hepatic metabolism among members of the SOCS family.

## 4. SOCS3 and Liver Diseases

The SOCS3 protein acts as a negative regulator of IL-6 signaling. SOCS3-deficient mice die during embryogenesis due to placental insufficiency [74]. Riehle et al. reported that SOCS3 hepatocyte-specific knock-out mice demonstrated increased proliferation and liver mass restoration after PH as compared with littermate controls [10]. They also reported that, in the absence of SOCS3, STAT3 phosphorylation was prolonged and activation of the mitogenic extracellular signal-regulated kinase 1/2 (ERK1/2) was upregulated after PH. Aoyama et al. investigated SOCS3 mRNA in pioglitazone-treated obese diabetic KK-Ay mice and found that pioglitazone prevented increases in STAT3 phosphorylation and SOCS3 mRNA after PH [75].

Yang et al. reported that high SOCS3 expression was associated with the presence of vascular invasion of HCC and poor overall survival in 87 HCC patients [76]. Zhang et al. reported that SOCS3 hypermethylation was significantly associated with poor clinical outcomes in HBV-infected HCC patients [77]. Niwa et al. found that the SOCS3 gene was aberrantly methylated in three of 10 human HCC cell lines and reported that SOCS3 negatively regulated cell growth and cell motility by inhibiting the JAK/STAT pathway in HCC cells [78]. The loss of SOCS3 by associated DNA methylation is favorable to cell growth and migration. 

Chronic IL-6 injection has been shown to selectively impair hepatic insulin signaling in mice [79]. Kim et al. demonstrated that IL-6 treatment reduced insulin ability to suppress hepatic glucose production [80]. Deletion of IL-6 improved hepatic insulin action in obese mice [81]. Overexpression of SOCS3 in the liver induced insulin resistance in mice and SOCS3 deletion improved insulin sensitivity [26,37]. Th SOCS3 seems to have a dual role in insulin activity in the liver. A short-term decrease of SOCS3 in the liver improves insulin sensitivity; however, long-term suppression of SOCS3 induces metabolic syndromes such as hyperglycemia and obesity [82].

Recently, an IL-6 receptor antagonist (tocilizumab) has been proposed as a treatment for coronavirus disease 2019 (COVID-19). Somers et al. reported that tocilizumab was associated with a 45% reduction in hazard of death (HR 0.55, 95% CI 0.33–0.94) by inverse probability of treatment weighting adjustment model [83]. Although tocilizumab was associated with an increased rate of superinfection, the superinfection was not significantly associated with 28-day fatality rate. In a phase III randomized controlled trial with 452 patients, mortality at Day 28 was 19.7% in the tocilizumab group and 19.4% in the placebo group, and the use of tocilizumab did not result in better clinical outcomes [84]. In a global phase III clinical trial with 389 patients, Salama et al. reported that tocilizumab reduced the risk of progression to mechanical ventilation or death, but it did not improve survival [85]. The timing of administration or a combination should be investigated further. 

## 5. Other SOCS and Liver Diseases

Yoshimura et al. searched for cytokine-responsive genes and identified a novel gene, cytokine-inducible SH2-containing protein (CIS), induced in hematopoietic cells by IL-2, IL-3, GM-CSF, and erythropoietin [14]. The CIS-1 transgenic overexpression mice showed defects in growth, mammary gland development, and T-cell response, indicating CIS was involved in GH signaling, prolactin signaling, and IL-2 signaling [86]. Hu et al. reported that two SNPs in the CIS-1 gene (rs414171 and rs2239751) were associated with persistent HBV infection [41].

Both SOCS4 and SOCS5 act as inhibitors of epidermal growth factor receptor (EGFR) signaling [38]. Calvisi et al. proposed two distinct subclasses of HCC associated with survival length and found SOCS4 and SOCS5 were upregulated in HCC with better outcome subclasses [87,88]. Sanchez-Mejias et al. reported a suppressive role of SOCS5 in HCC and found SOCS5 to be a target of miR-18 and miR-25 [89].

The function of SOCS6 is still largely unknown. Yoon et al. reported that mRNA and protein levels of SOCS6 were downregulated in HCC tissues [39]. Qiu et al. analyzed mRNA and protein levels of SOCS2 and SOCS6 in 106 HCC patients and found that both SOCS2 and SOCS6 downregulation were independent prognostic factors for poor overall survival (*p* = 0.008 and 0.01, respectively) [90].

Krebs et al. generated Socs7−/− mice, and they were 7–10% smaller than their wild type littermates, and half of them died within 15 weeks as a result of hydrocephalus [40]. Banks et al. reported that SOCS7 null mice exhibited increased growth of pancreatic islets with mildly increased fasting insulin levels and hypoglycemia [91]. It is of interest that SOCS2 null and SOCS7 null had an opposite phenotype in body growth. Fu et al. found that SOCS7 interacted with protein tyrosine phosphatase non-receptor type 14 (PTPN14) and blocked the NF-κB signaling pathway by preventing the activity of the inhibitor of NFκB kinase (IKK) complex in the acute liver failure mouse model [92]. 

## 6. Therapeutic Implications

The SOCS family has a crucial role in cell proliferation, metabolism, and the immune system. The family has a potential therapeutic role in cancer therapy. Waiboci et al. developed a small tyrosine kinase inhibitor peptide (Tkip) and it blocked phosphorylation of STAT1 and functioned as an antagonist of SOCS1 [93]. Flowers et al. reported that Tkip had an inhibitory effects on several prostate cancer cell lines [94]. There are several reports target the JAK/STAT pathway in HCC. Wilson et al. evaluated the antitumor effects of JAK inhibitor, ruxolitinib, and found that ruxolitinib inhibited JAK/STAT signaling and reduced the cell proliferation and colony formation of HCC cell lines HuH7, SNU182, and SNU423 [95].

Immuno-oncology is an emerging novel and pivotal cancer therapy through the stimulation of the immune system [96,97]. The combination of atezolizumab, an immune checkpoint inhibitor and bevacizumab, an anti-vascular endothelial growth factor neutralizing antibody, is now the first-line therapy for advanced HCC [98]. Overall survival rate at 12-month with atezolizumab-bevacizumab was 67.2% (95% CI, 61.3–73.1) and was 54.6% (95% CI, 45.2–64.0) with sorafenib. The hazard ratio for death was 0.58 (95% CI, 0.42–0.79; *p* < 0.001) [98]. There are almost 30 ongoing phase III trials testing immunotherapies for HCC [99]. Because SOCS1 is a regulator of interferon signaling, further studies are required to elucidate its role in cancer immunotherapy.

## 7. Conclusions

In the past two decades, since the discovery of SOCS1, there has been significant improvement in the understanding of the SOCS family of proteins. This has led to an understanding of the critical roles that SOCS proteins play in signaling pathways in liver disease, HCC development, and liver regeneration. Further study of these pathways may help elucidate mechanisms of carcinogenesis as well as enhance strategies for improving therapeutic options for patients with HCC. Further research should be employed to clarify the systemic feedback system and downstream regulation of cytokine signaling.

## Figures and Tables

**Table 1:** A summary of the SOCS family in liver diseases.

SOCS	Related Pathway	Significance	References
SOCS1	HGF	Decrease hepatocyte proliferation	[34]
	IFN-gamma	Inhibit antiviral activity	[35]
SOCS2	GH/JAK2/STAT5/IGF-1	Modulate liver regeneration	[9]
		Knock-out mice have gigantism	
SOCS3	STAT3	Decrease hepatocyte proliferation	[10]
	insulin	Induce insulin resistance	[36,37]
	G-CSF		
SOCS4	EGFR	The patients with upregulated SOCS4 have better clinical outcomes	[38]
		Reduce EGFR protein level	
SOCS5	EGFR	Reduce EGFR protein level	[38]
SOCS6	IGF-1	mRNA and protein levels are downregulated in HCC tissue	[39]
SOCS7	IGF-1	Knock-out mice are smaller than wild type mice.	[40]
CIS	STAT5	Inhibit GH	
		Associated with persistent hepatitis B infection	[41]

Abbreviations: SOCS, suppressor of cytokine signaling; HGF, hepatocyte growth factor; IFN, interferon; GH, growth hormone; JAK, Janus kinase; STAT, signal transducer and activator of transcription; IGF, insulin like growth factor; G-CSF, granulocyte-colony stimulating factor; EGFR, epidermal growth factor receptor; HCC, hepatocellular carcinoma.

## References

[1] Sung H., Ferlay J., Siegel R.L., Laversanne M., Soerjomataram I., Jemal A., Bray F. (2021). Global Cancer Statistics 2020: GLOBOCAN Estimates of Incidence and Mortality Worldwide for 36 Cancers in 185 Countries. CA Cancer J. Clin..

[2] Fitzmaurice C., Allen C., Barber R.M., Barregard L., Bhutta Z.A., Brenner H., Dicker D.J., Chimed-Orchir O., Dandona R., Dandona L. (2017). Global, Regional, and National Cancer Incidence, Mortality, Years of Life Lost, Years Lived with Disability, and Disability-Adjusted Life-Years for 32 Cancer Groups, 1990 to 2015: A Systematic Analysis for the Global Burden of Disease Study Global Burden. JAMA Oncol..

[3] Masuzaki R., Yoshida H., Tateishi R., Shiina S., Omata M. (2008). Hepatocellular Carcinoma in Viral Hepatitis: Improving Standard Therapy. Best Pract. Res. Clin. Gastroenterol..

[4] Heimbach J.K., Kulik L.M., Finn R.S., Sirlin C.B., Abecassis M.M., Roberts L.R., Zhu A.X., Murad M.H., Marrero J.A. (2018). AASLD Guidelines for the Treatment of Hepatocellular Carcinoma. Hepatology.

[5] Galle P.R., Forner A., Llovet J.M., Mazzaferro V., Piscaglia F., Raoul J.L., Schirmacher P., Vilgrain V. (2018). EASL Clinical Practice Guidelines: Management of Hepatocellular Carcinoma. J. Hepatol..

[6] Omata M., Cheng A.L., Kokudo N., Kudo M., Lee J.M., Jia J., Tateishi R., Han K.H., Chawla Y.K., Shiina S. (2017). Asia–Pacific Clinical Practice Guidelines on the Management of Hepatocellular Carcinoma: A 2017 Update. Hepatol. Int..

[7] Pinna A.D., Yang T., Mazzaferro V., De Carlis L., Zhou J., Roayaie S., Shen F., Sposito C., Cescon M., Di Sandro S. (2018). Liver Transplantation and Hepatic Resection Can Achieve Cure for Hepatocellular Carcinoma. Ann. Surg..

[8] Makuuchi M., Takayasu K., Takuma T., Yamazaki S., Hasegawa H., Nishiura S., Shimamura Y. (1984). Preoperative Transcatheter Embolization of the Portal Venous Branch for Patients Receiving Extended Lobectomy Due to the Bile Duct Carcinoma. J. Jpn. Pract. Surg. Soc..

[9] Masuzaki R., Zhao S., Valerius M.T., Tsugawa D., Oya Y., Ray K.C., Karp S.J. (2016). SOCS2 Balances Metabolic and Restorative Requirements during Liver Regeneration. J. Biol. Chem..

[10] Riehle K.J., Campbell J.S., McMahan R.S., Johnson M.M., Beyer R.P., Bammler T.K., Fausto N. (2008). Regulation of Liver Regeneration and Hepatocarcinogenesis by Suppressor of Cytokine Signaling 3. J. Exp. Med..

[11] Krebs D.L., Hilton D.J. (2001). SOCS Proteins: Negative Regulators of Cytokine Signaling. Stem Cells.

[12] Starr R., Willson T.A., Viney E.M., Murray L.J.L., Rayner J.R., Jenkins B.J., Gonda T.J., Alexander W.S., Metcalf D., Nicola N.A. (1997). A Family of Cytokine-Inducible Inhibitors of Signalling. Nature.

[13] Yoshimura A., Naka T., Kubo M. (2007). SOCS Proteins, Cytokine Signalling and Immune Regulation. Nat. Rev. Immunol..

[14] Yoshimura A., Ohkubo T., Kiguchi T., Jenkins N.A., Gilbert D.J., Copeland N.G., Hara T., Miyajima A. (1995). A Novel Cytokine-Inducible Gene CIS Encodes an SH2-Containing Protein That Binds to Tyrosine-Phosphorylated Interleukin 3 and Erythropoietin Receptors. EMBO J..

[15] Endo T.A., Masuhara M., Yokouchi M., Suzuki R., Sakamoto H., Mitsui K., Matsumoto A., Tanimura S., Ohtsubo M., Misawa H. (1997). A New Protein Containing an SH2 Domain That Inhibits JAK Kinases. Nature.

[16] Naka T., Narazaki M., Hirata M., Matsumoto T., Minamoto S., Aono A., Nishimoto N., Kajita T., Taga T., Yoshizaki K. (1997). Structure and Function of a New STAT-Induced STAT Inhibitor. Nature.

[17] Chen X.P., Losman J.A., Rothman P. (2000). SOCS Proteins, Regulators of Intracellular Signaling. Immunity.

[18] Naka T., Fujimoto M. (2010). SOCS1, a Negative Regulator of Cytokine Signals and TLR Responses, in Human Liver Diseases. Gastroenterol. Res. Pract..

[19] Croker B.A., Kiu H., Nicholson S.E. (2008). SOCS Regulation of the JAK/STAT Signalling Pathway. Semin. Cell Dev. Biol..

[20] Kile B.T., Schulman B.A., Alexander W.S., Nicola N.A., Martin H.M.E., Hilton D.J. (2002). The SOCS Box: A Tale of Destruction and Degradation. Trends Biochem. Sci..

[21] Alexander W.S. (2002). Suppressors of Cytokine Signalling (SOCS) in the Immune System. Nat. Rev. Immunol..

[22] Kamura T., Maenaka K., Kotoshiba S., Matsumoto M., Kohda D., Conaway R.C., Conaway J.W., Nakayama K.I. (2004). VHL-Box and SOCS-Box Domains Determine Binding Specificity for Cul2-Rbx1 and Cul5-Rbx2 Modules of Ubiquitin Ligases. Genes Dev..

[23] Yao R., Ma Y.L., Liang W., Li H.H., Ma Z.J., Yu X., Liao Y.H. (2012). MicroRNA-155 Modulates Treg and Th17 Cells Differentiation and Th17 Cell Function by Targeting SOCS1. PLoS ONE.

[24] Chen X.P., Losman J.A., Cowan S., Donahue E., Fay S., Vuong B.Q., Nawijn M.C., Capece D., Cohan V.L., Rothman P. (2002). Pim Serine/Threonine Kinases Regulate the Stability of Socs-1 Protein. Proc. Natl. Acad. Sci. USA.

[25] Sharma J., Larkin J. (2019). Therapeutic Implication of SOCS1 Modulation in the Treatment of Autoimmunity and Cancer. Front. Pharmacol..

[26] Yoshikawa H., Matsubara K., Qian G.-S., Jackson P., Groopman J.D., Manning J.E., Harris C.C., Herman J.G. (2001). SOCS-1, a Negative Regulator of the JAK/STAT Pathway, Is Silenced by Methylation in Human Hepatocellular Carcinoma and Shows Growth-Suppression Activity. Nat. Genet..

[27] Nagai H., Naka T., Terada Y., Komazaki T., Yabe A., Jin E., Kawanami O., Kishimoto T., Konishi N., Nakamura M. (2003). Hypermethylation Associated with Inactivation of the SOCS-1 Gene, a JAK/STAT Inhibitor, in Human Hepatoblastomas. J. Hum. Genet..

[28] Galm O., Yoshikawa H., Esteller M., Osieka R., Herman J.G. (2003). SOCS-1, a Negative Regulator of Cytokine Signaling, Is Frequently Silenced by Methylation in Multiple Myeloma. Blood.

[29] Chen C.Y., Tsay W., Tang J.L., Shen H.L., Lin S.W., Huang S.Y., Yao M., Chen Y.C., Shen M.C., Wang C.H. (2003). SOCS1 Methylation in Patients with Newly Diagnosed Acute Myeloid Leukemia. Genes Chromosom. Cancer.

[30] Fukushima N., Sato N., Sahin F., Su G.H., Hruban R.H., Goggins M. (2003). Aberrant Methylation of Suppressor of Cytokine Signalling-1 (SOCS-1) Gene in Pancreatic Ductal Neoplasms. Br. J. Cancer.

[31] Kanda T., Goto T., Hirotsu Y., Masuzaki R., Moriyama M., Omata M. (2020). Molecular Mechanisms: Connections between Nonalcoholic Fatty Liver Disease, Steatohepatitis and Hepatocellular Carcinoma. Int. J. Mol. Sci..

[32] Chhabra Y., Lee C.M.M., Müller A.F., Brooks A.J. (2020). GHR Signalling: Receptor Activation and Degradation Mechanisms. Mol. Cell. Endocrinol..

[33] Sasi W., Sharma A.K., Mokbel K. (2014). The Role of Suppressors of Cytokine Signalling in Human Neoplasms. Mol. Biol. Int..

[34] Gui Y., Yeganeh M., Ramanathan S., Leblanc C., Pomerleau V., Ferbeyre G., Saucier C., Ilangumaran S. (2011). SOCS1 Controls Liver Regeneration by Regulating HGF Signaling in Hepatocytes. J. Hepatol..

[35] Vlotides G., Sörensen A.S., Kopp F., Zitzmann K., Cengic N., Brand S., Zachoval R., Auernhammer C.J. (2004). SOCS-1 and SOCS-3 Inhibit IFN-α-Induced Expression of the Antiviral Proteins 2,5-OAS and MxA. Biochem. Biophys. Res. Commun..

[36] Ueki K., Kondo T., Kahn C.R. (2004). Suppressor of Cytokine Signaling 1 (SOCS-1) and SOCS-3 Cause Insulin Resistance through Inhibition of Tyrosine Phosphorylation of Insulin Receptor Substrate Proteins by Discrete Mechanisms. Mol. Cell. Biol..

[37] Ueki K., Kondo T., Tseng Y.H., Kahn C.R. (2004). Central Role of Suppressors of Cytokine Signaling Proteins in Hepatic Steatosis, Insulin Resistance, and the Metabolic Syndrome in the Mouse. Proc. Natl. Acad. Sci. USA.

[38] Kario E., Marmor M.D., Adamsky K., Citri A., Amit I., Amariglio N., Rechavi G., Yarden Y. (2005). Suppressors of Cytokine Signaling 4 and 5 Regulate Epidermal Growth Factor Receptor Signaling. J. Biol. Chem..

[39] Yoon S., Yi Y.S., Kim S.S., Kim J.H., Park W.S., Nam S.W. (2012). SOCS5 and SOCS6 Have Similar Expression Patterns in Normal and Cancer Tissues. Tumor Biol..

[40] Krebs D.L., Metcalf D., Merson T.D., Voss A.K., Thomas T., Zhang J.G., Rakar S., O’Bryan M.K., Willson T.A., Viney E.M. (2004). Development of Hydrocephalus in Mice Lacking SOCS7. Proc. Natl. Acad. Sci. USA.

[41] Hu Z., Yang J., Wu Y., Xiong G., Wang Y., Yang J., Deng L. (2014). Polymorphisms in CISH Gene Are Associated with Persistent Hepatitis B Virus Infection in Han Chinese Population. PLoS ONE.

[42] Whittaker S., Marais R., Zhu A.X. (2010). The Role of Signaling Pathways in the Development and Treatment of Hepatocellular Carcinoma. Oncogene.

[43] Villanueva A., Newell P., Chiang D.Y., Friedman S.L., Llovet J.M. (2007). Genomics and Signaling Pathways in Hepatocellular Carcinoma. Semin. Liver Dis..

[44] Shibata T., Aburatani H. (2014). Exploration of Liver Cancer Genomes. Nat. Rev. Gastroenterol. Hepatol..

[45] JS L. (2015). The Mutational Landscape of Hepatocellular Carcinoma. Clin. Mol. Hepatol..

[46] Liau N.P.D., Laktyushin A., Lucet I.S., Murphy J.M., Yao S., Whitlock E., Callaghan K., Nicola N.A., Kershaw N.J., Babon J.J. (2018). The Molecular Basis of JAK/STAT Inhibition by SOCS1. Nat. Commun..

[47] Naka T., Tsutsui H., Fujimoto M., Kawazoe Y., Kohzaki H., Morita Y., Nakagawa R., Narazaki M., Adachi K., Yoshimoto T. (2001). SOCS-1/SSI-1-Deficient NKT Cells Participate in Severe Hepatitis through Dysregulated Cross-Talk Inhibition of IFN-γ and IL-4 Signaling in Vivo. Immunity.

[48] Okochi O., Hibi K., Sakai M., Inoue S., Takeda S., Kaneko T., Nakao A. (2003). Methylation-Mediated Silencing of SOCS-1 Gene in Hepatocellular Carcinoma Derived from Cirrhosis. Clin. Cancer Res..

[49] Yoshida T., Ogata H., Kamio M., Joo A., Shiraishi H., Tokunaga Y., Sata M., Nagai H., Yoshimura A. (2004). SOCS1 Is a Suppressor of Liver Fibrosis and Hepatitis-Induced Carcinogenesis. J. Exp. Med..

[50] Ko E., Kim S.J., Joh J.W., Park C.K., Park J., Kim D.H. (2008). CpG Island Hypermethylation of SOCS-1 Gene Is Inversely Associated with HBV Infection in Hepatocellular Carcinoma. Cancer Lett..

[51] Tam W. (2001). Identification and Characterization of Human BIC, a Gene on Chromosome 21 That Encodes a Noncoding RNA. Gene.

[52] Kawai T., Akira S. (2006). TLR Signaling. Cell Death Differ..

[53] Bala S., Petrasek J., Csak T., Catalano D., Kodys K., Mundkur S., Szabo G. (2012). MicroRNA-155 Regulates Inflammation in Alcoholic Liver Disease via Targeting SOCS1 and SHIP1 (54.15). J. Immunol..

[54] Bin Wahid S., Ain Q.U., Quraishi A., Wahid B. (2020). Clinical Correlation of Liver Function Tests with Suppression of Cytokine Signaling (SOCS1) Gene Expression in HCV Infected Patients: A Real-World Clinical Experience. J. Med. Virol..

[55] Davis G.L., Hoofnagle J.H. (1986). Interferon in Viral Hepatitis: Role in Pathogenesis and Treatment. Hepatology.

[56] Yeh M.-L., Huang J.-F., Yu M.-L., Chuang W.-L. (2020). Hepatitis b Infection: Progress in Identifying Patients Most Likely to Respond to Peginterferon Alfa. Expert Rev. Gastroenterol. Hepatol..

[57] Kanda T., Sasaki R., Masuzaki R., Takahashi H., Fujisawa M., Matsumoto N., Okamoto H., Moriyama M. (2020). Additive Effects of Zinc Chloride on the Suppression of Hepatitis A Virus Replication by Interferon in Human Hepatoma Huh7 Cells. In Vivo.

[58] Kang S., Myoung J. (2017). Host Innate Immunity against Hepatitis e Virus and Viral Evasion Mechanisms. J. Microbiol. Biotechnol..

[59] Platanias L.C. (2005). Mechanisms of Type-I- and Type-II-Interferon-Mediated Signalling. Nat. Rev. Immunol..

[60] Metz P., Dazert E., Ruggieri A., Mazur J., Kaderali L., Kaul A., Zeuge U., Windisch M.P., Trippler M., Lohmann V. (2012). Identification of Type I and Type II Interferon-Induced Effectors Controlling Hepatitis C Virus Replication. Hepatology.

[61] Suppiah V., Moldovan M., Ahlenstiel G., Berg T., Weltman M., Abate M.L., Bassendine M., Spengler U., Dore G.J., Powell E. (2009). IL28B Is Associated with Response to Chronic Hepatitis C Interferon-α and Ribavirin Therapy. Nat. Genet..

[62] Akhtar L.N., Benveniste E.N. (2011). Viral Exploitation of Host SOCS Protein Functions. J. Virol..

[63] Dalpke A., Heeg K., Bartz H., Baetz A. (2008). Regulation of Innate Immunity by Suppressor of Cytokine Signaling (SOCS) Proteins. Immunobiology.

[64] Song M.M., Shuai K. (1998). The Suppressor of Cytokine Signaling (SOCS) 1 and SOCS3 but Not SOCS2 Proteins Inhibit Interferon-Mediated Antiviral and Antiproliferative Activities. J. Biol. Chem..

[65] Kanda T., Lau G.K.K., Wei L., Moriyama M., Yu M.L., Chuang W.L., Ibrahim A., Lesmana C.R.A., Sollano J., Kumar M. (2019). APASL HCV Guidelines of Virus-Eradicated Patients by DAA on How to Monitor HCC Occurrence and HBV Reactivation. Hepatol. Int..

[66] Naz Z., Wahid B., Usman S., Saleem K., Rafique S., Ali A., Idrees M. (2018). Expression of SOCS1 and SOCS3 Genes in Interferon-Treated and Direct-Acting Antiviral Drugs-Treated Hepatitis C Patients. J. Interf. Cytokine Res..

[67] Metcalf D., Greenhalgh C.J., Viney E., Wilison T.A., Starr R., Nicola N.A., Hilton D.J., Alexander W.S. (2000). Gigantism in Mice Lacking Suppressor of Cytokine Signalling-2. Nature.

[68] Cui M., Sun J., Hou J., Fang T., Wang X., Ge C., Zhao F., Chen T., Xie H., Cui Y. (2016). The Suppressor of Cytokine Signaling 2 (SOCS2) Inhibits Tumor Metastasis in Hepatocellular Carcinoma. Tumor Biol..

[69] Ren W., Wu S., Wu Y., Liu T., Zhao X., Li Y. (2019). MicroRNA-196a/-196b Regulate the Progression of Hepatocellular Carcinoma through Modulating the JAK/STAT Pathway via Targeting SOCS2. Cell Death Dis..

[70] Li B., Feng W., Luo O., Xu T., Cao Y., Wu H., Yu D., Ding Y. (2017). Development and Validation of a Three-Gene Prognostic Signature for Patients with Hepatocellular Carcinoma. Sci. Rep..

[71] Zadjali F., Santana-Farre R., Vesterlund M., Carow B., Mirecki-Garrido M., Hernandez-Hernandez I., Flodström-Tullberg M., Parini P., Rottenberg M., Norstedt G. (2012). SOCS2 Deletion Protects against Hepatic Steatosis but Worsens Insulin Resistance in High-fat-diet-fed Mice. FASEB J..

[72] Emanuelli B., Macotela Y., Boucher J., Ronald Kahn C. (2008). SOCS-1 Deficiency Does Not Prevent Diet-Induced Insulin Resistance. Biochem. Biophys. Res. Commun..

[73] Sachithanandan N., Fam B.C., Fynch S., Dzamko N., Watt M.J., Wormald S., Honeyman J., Galic S., Proietto J., Andrikopoulos S. (2010). Liver-Specific Suppressor of Cytokine Signaling-3 Deletion in Mice Enhances Hepatic Insulin Sensitivity and Lipogenesis Resulting in Fatty Liver and Obesity. Hepatology.

[74] Roberts A.W., Robb L., Rakar S., Hartley L., Cluse L., Nicola N.A., Metcalf D., Hilton D.J., Alexander W.S. (2001). Placental Defects and Embryonic Lethality in Mice Lacking Suppressor of Cytokine Signaling 3. Proc. Natl. Acad. Sci. USA.

[75] Aoyama T., Ikejima K., Kon K., Okumura K., Arai K., Watanabe S. (2009). Pioglitazone Promotes Survival and Prevents Hepatic Regeneration Failure after Partial Hepatectomy in Obese and Diabetic KK-Ay Mice. Hepatology.

[76] Yang S.F., Yeh Y.T., Wang S.N., Hung S.C., Chen W.T., Huang C.H., Chai C.Y. (2008). SOCS-3 Is Associated with Vascular Invasion and Overall Survival in Hepatocellular Carcinoma. Pathology.

[77] Zhang X., You Q., Zhang X., Chen X. (2015). SOCS3 Methylation Predicts a Poor Prognosis in HBV Infection-Related Hepatocellular Carcinoma. Int. J. Mol. Sci..

[78] Niwa Y., Kanda H., Shikauchi Y., Saiura A., Matsubara K., Kitagawa T., Yamamoto J., Kubo T., Yoshikawa H. (2005). Methylation Silencing of SOCS-3 Promotes Cell Growth and Migration by Enhancing JAK/STAT and FAK Signalings in Human Hepatocellular Carcinoma. Oncogene.

[79] Klover P.J., Zimmers T.A., Koniaris L.G., Mooney R.A. (2003). Chronic Exposure to Interleukin-6 Causes Hepatic Insulin Resistance in Mice. Diabetes.

[80] Kim H.J., Higashimori T., Park S.Y., Choi H., Dong J., Kim Y.J., Noh H.L., Cho Y.R., Cline G., Kim Y.B. (2004). Differential Effects of Interleukin-6 and -10 on Skeletal Muscle and Liver Insulin Action In Vivo. Diabetes.

[81] Klover P.J., Clementi A.H., Mooney R.A. (2005). Interleukin-6 Depletion Selectively Improves Hepatic Insulin Action in Obesity. Endocrinology.

[82] Torisu T., Sato N., Yoshiga D., Kobayashi T., Yoshioka T., Mori H., Iida M., Yoshimura A. (2007). The Dual Function of Hepatic SOCS3 in Insulin Resistance in Vivo. Genes Cells.

[83] Somers E.C., Eschenauer G.A., Troost J.P., Golob J.L., Gandhi T.N., Wang L., Zhou N., Petty L.A., Baang J.H., Dillman N.O. (2020). Tocilizumab for Treatment of Mechanically Ventilated Patients With COVID-19. Clin. Infect. Dis..

[84] Rosas I.O., Bräu N., Waters M., Go R.C., Hunter B.D., Bhagani S., Skiest D., Aziz M.S., Cooper N., Douglas I.S. (2021). Tocilizumab in Hospitalized Patients with Severe Covid-19 Pneumonia. N. Engl. J. Med..

[85] Salama C., Han J., Yau L., Reiss W.G., Kramer B., Neidhart J.D., Criner G.J., Kaplan-Lewis E., Baden R., Pandit L. (2021). Tocilizumab in Patients Hospitalized with Covid-19 Pneumonia. N. Engl. J. Med..

[86] Matsumoto A., Seki Y., Kubo M., Ohtsuka S., Suzuki A., Hayashi I., Tsuji K., Nakahata T., Okabe M., Yamada S. (1999). Suppression of STAT5 Functions in Liver, Mammary Glands, and T Cells in Cytokine-Inducible SH2-Containing Protein 1 Transgenic Mice. Mol. Cell. Biol..

[87] Calvisi D.F., Ladu S., Gorden A., Farina M., Lee J.S., Conner E.A., Schroeder I., Factor V.M., Thorgeirsson S.S. (2007). Mechanistic and Prognostic Significance of Aberrant Methylation in the Molecular Pathogenesis of Human Hepatocellular Carcinoma. J. Clin. Investig..

[88] Lee J.S., Chu I.S., Heo J., Calvisi D.F., Sun Z., Roskams T., Durnez A., Demetris A.J., Thorgeirsson S.S. (2004). Classification and Prediction of Survival in Hepatocellular Carcinoma by Gene Expression Profiling. Hepatology.

[89] Sanchez-Mejias A., Kwon J., Chew X.H., Siemens A., Sohn H.S., Jing G., Zhang B., Yang H., Tay Y. (2019). A Novel SOCS5/MiR-18/MiR-25 Axis Promotes Tumorigenesis in Liver Cancer. Int. J. Cancer.

[90] Qiu X., Zheng J., Guo X., Gao X., Liu H., Tu Y., Zhang Y. (2013). Reduced Expression of SOCS2 and SOCS6 in Hepatocellular Carcinoma Correlates with Aggressive Tumor Progression and Poor Prognosis. Mol. Cell. Biochem..

[91] Banks A.S., Li J., McKeag L., Hribal M.L., Kashiwada M., Accili D., Rothman P.B. (2005). Deletion of SOCS7 Leads to Enhanced Insulin Action and Enlarged Islets of Langerhans. J. Clin. Investig..

[92] Fu B., Yin S., Lin X., Shi L., Wang Y., Zhang S., Zhao Q., Li Z., Yang Y., Wu H. (2020). PTPN14 Aggravates Inflammation through Promoting Proteasomal Degradation of SOCS7 in Acute Liver Failure. Cell Death Dis..

[93] Waiboci L.W., Ahmed C.M., Mujtaba M.G., Flowers L.O., Martin J.P., Haider M.I., Johnson H.M. (2007). Both the Suppressor of Cytokine Signaling 1 (SOCS-1) Kinase Inhibitory Region and SOCS-1 Mimetic Bind to JAK2 Autophosphorylation Site: Implications for the Development of a SOCS-1 Antagonist. J. Immunol..

[94] Flowers L.O., Subramaniam P.S., Johnson H.M. (2005). A SOCS-1 Peptide Mimetic Inhibits Both Constitutive and IL-6 Induced Activation of STAT3 in Prostate Cancer Cells. Oncogene.

[95] Wilson G.S., Tian A., Hebbard L., Duan W., George J., Li X., Qiao L. (2013). Tumoricidal Effects of the JAK Inhibitor Ruxolitinib (INC424) on Hepatocellular Carcinoma in Vitro. Cancer Lett..

[96] Binnewies M., Roberts E.W., Kersten K., Chan V., Fearon D.F., Merad M., Coussens L.M., Gabrilovich D.I., Ostrand-Rosenberg S., Hedrick C.C. (2018). Understanding the Tumor Immune Microenvironment (TIME) for Effective Therapy. Nat. Med..

[97] Gajewski T.F., Schreiber H., Fu Y.X. (2013). Innate and Adaptive Immune Cells in the Tumor Microenvironment. Nat. Immunol..

[98] Finn R.S., Qin S., Ikeda M., Galle P.R., Ducreux M., Kim T.-Y., Kudo M., Breder V., Merle P., Kaseb A.O. (2020). Atezolizumab plus Bevacizumab in Unresectable Hepatocellular Carcinoma. N. Engl. J. Med..

[99] Llovet J.M., Castet F., Heikenwalder M., Maini M.K., Mazzaferro V., Pinato D.J., Pikarsky E., Zhu A.X., Finn R.S. (2021). Immunotherapies for Hepatocellular Carcinoma. Nat. Rev. Clin. Oncol..

